# Can cellular automata be a representative model for visual perception dynamics?

**DOI:** 10.3389/fncom.2013.00130

**Published:** 2013-10-01

**Authors:** Maryam Beigzadeh, Seyyed Mohammad R. Hashemi Golpayegani, Shahriar Gharibzadeh

**Affiliations:** Department of Biomedical Engineering, Amirkabir University of TechnologyTehran, Iran

**Keywords:** cellular automata, visual perception modeling, complex systems, chaos and nonlinear dynamics, EEG

“Cellular automata (CA) are mathematical models for systems in which many simple components act together to produce complicated patterns of behavior” (Packard and Wolfram, 1985). Applying the CA theoretical framework in the field of neuroscience has shown successful results in the interpretation of some cognitive aspects (Adams et al., 1992;Pashaie and Farhat, 2009); Kozma and Puljic, 2013; Lopez-Ruiz and Fournier-Prunaret, 2013; Mattei, 2013). In this short analysis, we suggest that CA can be a very reasonable tool to model both dynamical and structural aspects of visual perception. As Wolfram declared in his book, A New Kind of Science, visual perception is a kind of modeling and reducing the input visual sensory data into a more summary but still informative representation in the brain (Wolfram, 2002).

Studying the visual system can be very useful, because as already previously demonstrated, “The visual system has the most complex neural circuitry of all the sensory systems (Kandel et al., 2000)” and at least 20% of human cerebral cortex is related to the visual part (Olshausen, 2002). Additionally, trying to understand visual perception may lead us to a better understanding of how other cognitive processes in the brains work.

It has already been demonstrated that brain dynamics (which are reflected in EEG, MEG, and ECoG signals) are inherently chaotic (Freeman, 1991). As we perceive different sensory information (i.e., images, sounds, odors, etc.) and recognize different patterns, these dynamical processes tend to turn into a more regular pattern. This stage has been referred by other researchers as: “the transients between gas-like randomness and liquid-like order (Kozma et al., 2012).” According to such paradigm, each stimulus would tend to lead the system to its own “liquid-like attractor” which is different from the other one. So, after the sensorial stimuli, the brain dynamics would exhibit a temporary switching between these different states.

But what would be the advantage of using such CA model? There are millions of neurons in the visual system that are highly interactive, each one demonstrating its own complex behavior. Their combined and integrated functions lead to the overall process of perception. The CA framework provides a model, in which a collection of many interactive agents (cells) relate to each other according to specific “interaction rules” in space and time. The number of agents, their dynamical properties, and their interactions with each other, determine which kind of behavior (chaotic, periodic, etc.) the CA will adopt.

Compared to other alternative multi-agent modeling tools (such as artificial neural networks), in CA the researcher is able to determine the local behavior of individuals as well as their interaction rules and connectivity patterns, both locally and globally in space. In CA model it is also possible to analyze the behavior of the system from both the micro to macro levels. But how could the analysis of the space properties of CA make visual perception modeling more realistic? It has already been demonstrated that, in the visual system (at least in the primary processing areas such as V1) there are specialized cells which, because of their own specific structure and function, become more sensitive to specific properties of the perceived visual scene (such as image edges, textures, orientation, spatial frequencies) that are inherently space related features.

In such sense, CA would fit as a very appropriate model, as it exhibits close theoretical similarities with other methods which use graph theory and small world networks analysis (Sporns, 2006; Stam and Reijneveld, 2007). Additionally, it has already been suggested that probabilistic CA can be successfully employed to model the olfactory perception (Kozma et al., 2012). Nevertheless, using CA for modeling visual perception from the dynamical and structural standpoints has not yet been reported before, although CA has already been used for modeling simpler visual-related tasks, such as retina function, or as a computational tool for implementing image processing tasks in computer vision applications (like edge detection, texture detection, noise reduction, etc.) (Wolfram, 2002; Dhillon, 2012). In this short commentary we defend that CA can be used as a holistic model for the integration of local visual aspects in a broader multimodal integration of the global aspects of visual perception.

One possible strategy in order to implement such paradigm would be to use specific objective measures (such as the number of active neurons, or the mean activation value of a specific network), and afterwards attempt to match the behavior of such time series (by comparing its phase space and strange attractors) with real EEG recordings related to specific visual tasks (such as the classic “face/non face discrimination”).

In summary, the dynamic behavior of CA has been shown to be a power tool for modeling several types of neuronal activity and we believe that it can be successfully used to study global features of visual perception. In fact, future studies on this area may be able to demonstrate how perceptual deficits commonly observed in clinical practice (such as face recognition deficits in autistic patients) may be represented by a change in the basic parameters of CA models of visual representation.
